# Second-tier genetics improves newborn screening accuracy for SCID and other T cell deficiencies

**DOI:** 10.70962/jhi.20260031

**Published:** 2026-07-16

**Authors:** Annelotte J. Duintjer, Maartje Blom, Robbert G.M. Bredius, Ingrid Pico-Knijnenburg, Adinda Heuperman, Hennie Hodemaekers, Sandra Imholz, Martijn E.T. Dollé, Ruben B. Brandsema, Lisette van de Corput, Stefanie S. Henriet, Taco W. Kuijpers, Joris M. van Montfrans, Clementien L. Vermont, Gijs T.J. van Well, Evelien Zonneveld-Huijssoon, Els Voorhoeve, Mariëlle E. van Gijn, Mirjam van der Burg

**Affiliations:** 1 Department of Pediatrics, Laboratory for Pediatric Immunology, Willem-Alexander Children’s Hospital, Leiden University Medical Center, Leiden, Netherlands; 2 Department of Pediatrics, Willem-Alexander Children’s Hospital, Leiden University Medical Center, Leiden, Netherlands; 3 Centre for Health Protection, Dutch National Institute for Public Health and the Environment, Bilthoven, Netherlands; 4 Department of Pediatrics, Infectious Diseases and Immunology, Beatrix Children’s Hospital, University Medical Center Groningen, University of Groningen, Groningen, Netherlands; 5 Central Diagnostic Laboratory, University Medical Center Utrecht, Utrecht, Netherlands; 6 Department of Pediatric Immunology and Infectious Diseases, Amalia Children’s Hospital, Radboud University Medical Center, Nijmegen, Netherlands; 7 Department of Pediatric Immunology, Rheumatology and Infectious Diseases, Emma Children’s Hospital, Amsterdam University Medical Center, Amsterdam, Netherlands; 8 Department of Pediatric Immunology and Infectious Diseases, Wilhelmina Children’s Hospital, University Medical Center Utrecht, Utrecht, Netherlands; 9 Department of Pediatric Immunology and Infectious Diseases, Sophia Children’s Hospital, Erasmus MC, University Medical Center, Rotterdam, Netherlands; 10 Department of Pediatrics, Division of Infectious Diseases and Immunology, MosaKids Children’s Hospital, Maastricht University Medical Center, Maastricht, Netherlands; 11 Department of Genetics, University of Groningen, University Medical Center Groningen, Groningen, Netherlands; 12 Department of Human Genetics, Amsterdam University Medical Center, Amsterdam, Netherlands

## Abstract

Newborn screening (NBS) based on quantifying T cell receptor excision circles (TRECs) is highly sensitive for detecting severe combined immunodeficiency (SCID), but frequently results in false-positive referrals. These referrals contribute to parental distress, increased clinical workload, additional interventions, and costs. To increase the positive predictive value (PPV) for SCID and other T cell deficiencies with a genetic cause, we evaluated next-generation sequencing (NGS) as second-tier testing after TREC analysis. Targeted sequencing was performed on dried blood spots of 68 newborns referred from TREC-based NBS, and results were integrated with collected long-term follow-up data. A safety net algorithm was applied to maintain high sensitivity for SCID by directly referring newborns with profoundly reduced TRECs, increasing the PPV from 22% to 55% without missing patients with severe immunological phenotypes. Therefore, the clinical impact of not identifying non-severe T cell lymphopenia patients without a genetic diagnosis appears limited. These results demonstrate that second-tier NGS in TREC-based NBS improves screening accuracy for SCID and other T cell deficiencies.

## Teaser

This study demonstrates that second-tier genetics improves newborn screening accuracy for SCID and other T cell deficiencies. A safety net algorithm maintains high sensitivity for SCID while substantially increasing the PPV from 22.1% to 55.2%.

## Introduction

Newborn screening (NBS) for severe combined immunodeficiency (SCID) has been increasingly adopted worldwide. Detecting SCID through NBS allows early initiation of preventive measures and curative treatment with hematopoietic stem cell transplantation or gene therapy at a pre-symptomatic stage, leading to improved clinical outcomes and survival (1, 2, 3, 4).

SCID encompasses a group of monogenic disorders causing absent or dysfunctional T cells, often associated with aberrant development of other lymphocyte lineages as well (5, 6). NBS for SCID is performed using quantitative PCR (qPCR) to quantify T cell receptor excision circles (TRECs) in dried blood spots (DBSs), which serve as a molecular biomarker for T cell maturation in the thymus (7, 8). TREC quantification is highly sensitive for detecting T cell lymphopenia and, consequently, for identifying SCID. However, it more frequently results in secondary findings, as multiple other conditions also present with low T cells at birth (8, 9, 10, 11). Besides SCID, other inborn errors of immunity (IEIs), such as cartilage hair hypoplasia (*RMRP* gene) and ataxia telangiectasia (*ATM* gene), can also present with low TRECs (6, 12, 13). Notably, since the introduction of TREC-based NBS, an increasing number of patients with T cell lymphopenia carrying heterozygous *FOXN1* variants have been found (14). Genetic syndromes can also be associated with low TRECs, including 22q11.2 deletion syndrome, trisomy 21, and Noonan syndrome (12, 13, 15). Nongenetic secondary findings can include idiopathic T cell lymphopenia (ITCL), preterm birth or low birth weight, and various reversible conditions such as neonatal infections, congenital anomalies, and maternal immunosuppressant use (12, 13, 16, 17). Finally, false-positive TREC results can occur where diagnostic immunophenotyping reveals normal lymphocyte subsets, but no clinical cause for the low TRECs is identified (10). In screening programs that define only SCID as the screening target, the TREC assay consequently has a low positive predictive value (PPV). Some programs apply a broader definition for the screening target, such as SCID and severe T cell lymphopenia (18), which results in a higher PPV.

Among these secondary findings, it is important to distinguish actionable findings, where early intervention due to early detection improves clinical outcomes, from non-actionable findings, where early detection does not enable intervention or where intervention does not lead to health gain (19). Non-actionable findings contribute not only to increased workload, additional medical interventions, and healthcare costs but also to significant emotional strain on parents, often causing stress, uncertainty, and anxiety (20). To decrease non-actionable findings and false-positives, second-tier tests after TREC analysis have been proposed, including epigenetic immune cell counting to quantify relative CD3^+^ T cell counts and an alternative TREC qPCR assay. Although these methods were effective, the impact on the referral rate was limited and may miss potentially actionable genetic findings such as *FOXN1* haploinsufficiency and 22q11.2 deletion syndrome (21).

Second-tier next-generation sequencing (NGS) could substantially reduce referrals in TREC-based NBS while maintaining high sensitivity for genetic findings. This approach could enable a rapid genetic diagnosis not only for patients with SCID but also for other IEIs presenting with low TRECs and T cell lymphopenia (22, 23). In this study, we evaluated targeted NGS as a second-tier test after TREC analysis and integrated the results with retrospective patient follow-up data to assess the clinical impact on reducing non-actionable secondary findings.

## Results

### Technical performance

**Figure 1.**
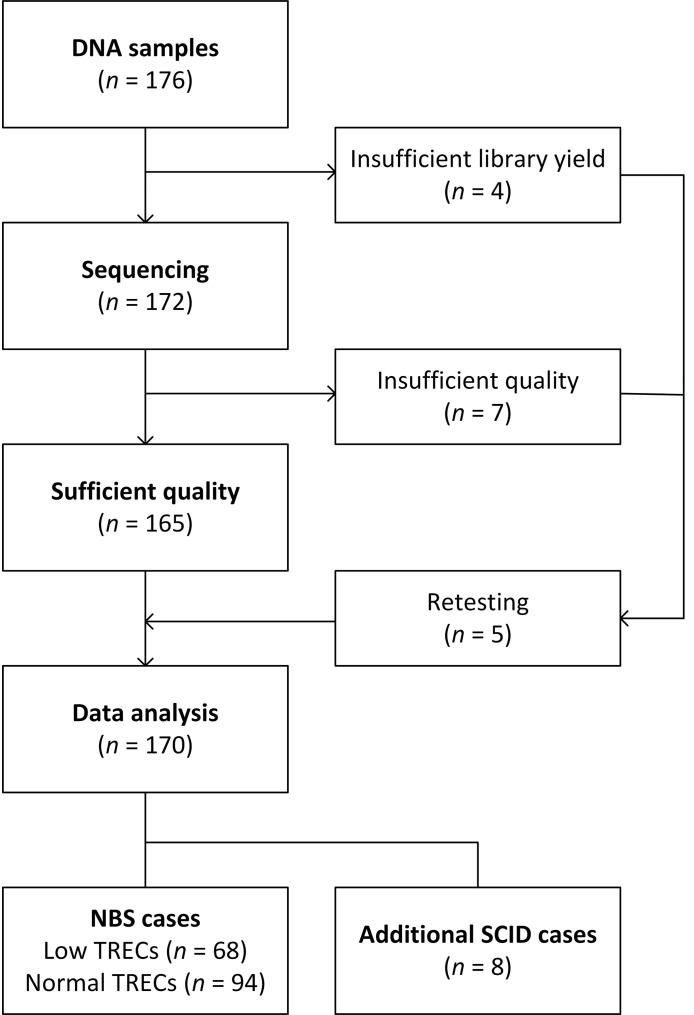
Sample processing.

**Figure S1.**
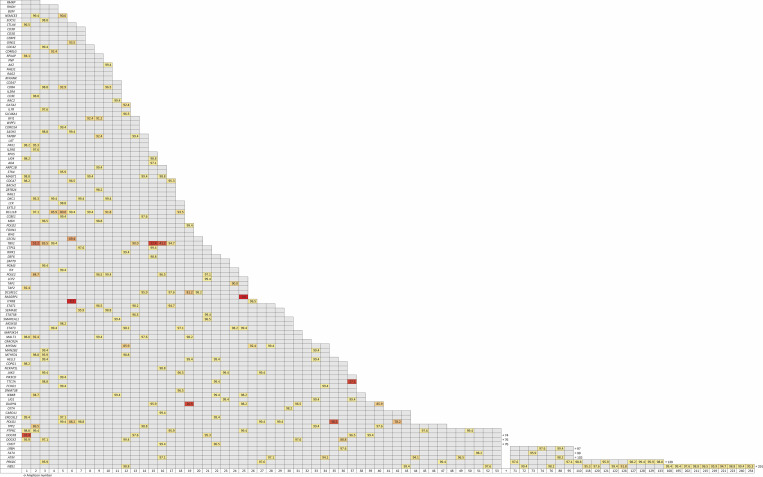
**Percentage of samples with sufficient read depth per amplicon.** For each gene (y-axis) and amplicon (x-axis), the percentage of samples (*n* total = 170) with a read depth of ≥20× is shown. Only values below 100% are displayed; grey cells indicate amplicons for which 100% of samples achieved ≥20× read depth. For genes with >53 amplicons, results are summarized with amplicons not displayed being 100%.

Libraries were prepared for 176 DNA samples; 68 with low TRECs in NBS (≤10 copies/3.2 mm DBS punch), 100 with normal TRECs in NBS, and 8 additional DBS samples from SCID patients (Fig. 1). Four samples were excluded due to insufficient library yield, leaving 172 (97.7%) for sequencing. Of these, 165 (95.9%) achieved sufficient coverage (≥95% of the target region with 20× read depth). All failed low-TREC NBS samples (*n* = 3) were retested, along with NBS samples with normal TRECs, resulting in 170 samples for data analysis. Overall, 92.7% of amplicons (*n* total = 3,003) had ≥20× read depth across all analyzed samples (*n* = 170) (Fig. S1). Based on logged individual experiences, the estimated time to obtain sequencing data was 3 days, comprising 1 h for DNA isolation, 1.5 days for library preparation (including overnight PCR), and overnight sequencing. Including data analysis and reporting, the turnaround time would be approximately 4 days, depending on how second-tier NGS is implemented into routine screening practice.

### Variant interpretation

**Figure 2.**
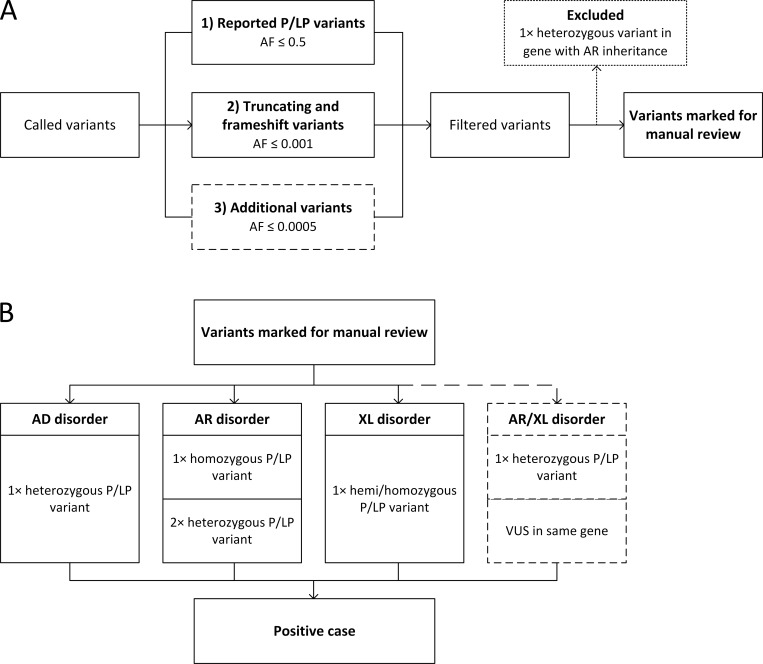
**Variant filtering and reporting. (A and B)** Flowcharts of the (A) applied variant filtering and (B) variant reporting strategy. A detailed overview of the applied filters is provided in Table S2. Heterozygous pathogenic (P) or likely pathogenic (LP) variants in genes with AR and XL inheritance were only reported when an additional VUS was identified, as indicated by the dashed box (B). AD, autosomal dominant.

From the 162 NBS cases included in the data analysis, 124 (76.5%) were directly screen negative, indicating that there were no filtered variants marked for manual review (Fig. 2). In the remaining 38 cases, which included 25 of the low-TREC cases (36.8%) and 13 of the normal-TREC cases (13.8%), 50 variants were filtered and manually reviewed for pathogenicity. Of the reviewed variants, 22 were classified as a variant of uncertain significance (VUS), including 9 variants not reported in the assessed variant databases (Table S1). A VUS was found in 12 of the low-TREC cases (17.6%) and 10 of the normal-TREC cases (10.6%).

All pathogenic variants in the 15 SCID and IEI patients from NBS were confirmed (Table 1). A detailed overview of the variants detected in cases 1–10 is provided in Table S3. An additional heterozygous *TBX1* deletion was found in a low-TREC case (case 52), in which T cell levels normalized after repeat immunophenotyping, and low TRECs were attributed to low birth weight. No genetic diagnostics were performed, so this finding could not be confirmed from the existing clinical data and was therefore reported to the treating physician. No pathogenic or likely pathogenic variants were found in the NBS cases with normal TRECs. In all NBS cases (*n* = 162), no carriers of single heterozygous pathogenic or likely pathogenic variants in genes with autosomal recessive (AR) inheritance were identified.

**Table 1.** Positive NBS cases

Case no.	Gene (MOI)	Variant	Zygosity	TRECs^a^	Phenotype
1	*RAG1* (AR)	NM_000448.3: c.519del p.(Glu174SerfsTer27)	Homozygous	0	SCID
2	*RAG1* (AR)	NM_000448.3: c.1331C>T p.(Ala444Val)	Homozygous	0	SCID
3	*RAG1* (AR)	NM_000448.3: c.2095C>T p.(Arg699Trp) and c.2974A>G p.(Lys992Glu)	Compound heterozygous	0	SCID
4	*IL2RG* (XL)	NM_000206.3: c.298C>T p.(Gln100Ter)	Hemizygous	0	SCID
5	*IL2RG* (XL)	NM_000206.3: c.190G>A p.(Val64Met)	Hemizygous	0	SCID
6	*FOXN1* (AD/AR)	NM_003593.3: c.831-2A>G p.(?)	Heterozygous	>2 and ≤10	*FOXN1* haploinsufficiency
7	*FOXN1* (AD/AR)	NM_003593.3: c.143del p.(Cys48SerfsTer254)	Heterozygous	>2 and ≤10	*FOXN1* haploinsufficiency
8	*FOXN1* (AD/AR)	NM_003593.3: c.1079T>C p.(Leu360Pro)	Heterozygous	≤2	*FOXN1* haploinsufficiency
9	*RMRP* (AR)	NR_003051.3: n.147G>A and n.-32_1dup	Compound heterozygous	≤2	Cartilage hair hypoplasia
10	*ATM* (AR)	NM_000051.4:c.5979_5983del p.(Ser1993ArgfsTer23) and c.7875_7876delinsGC p.(Asp2625_Ala2626delinsGluPro)	Compound heterozygous	>2 and ≤10	Ataxia telangiectasia
11	*TBX1* (AD)	*TBX1* deletion	Heterozygous	≤2	22q11.2 deletion syndrome
12	*TBX1* (AD)	*TBX1* deletion	Heterozygous	≤2	22q11.2 deletion syndrome
13	*TBX1* (AD)	*TBX1* deletion	Heterozygous	>2 and ≤10	22q11.2 deletion syndrome
14	*TBX1* (AD)	*TBX1* deletion	Heterozygous	>2 and ≤10	22q11.2 deletion syndrome
15	*TBX1* (AD)	*TBX1* deletion	Heterozygous	>2 and ≤10	22q11.2 deletion syndrome
52	*TBX1* (AD)	*TBX1* deletion	Heterozygous	>2 and ≤10	Unknown

AD, autosomal dominant; MOI, mode of inheritance. Detected variants, TREC results, and clinical phenotype of positive cases identified by second-tier NGS in the analyzed NBS samples (*n* total = 162).

a Copies/3.2 mm DBS punch (ImmunoIVD).

Among the additionally included eight SCID cases, seven were confirmed, including a homozygous copy number variation (CNV) involving deletion of exons 1–3 in the *DCLRE1C* gene (Table S4). One variant was missed in a compound heterozygous *RAG2* case (DBS-5). This variant was not reported in ClinVar and was filtered out due to not passing the variant call format quality filter (Table S2).

### Reduction in referrals

**Figure 3.**
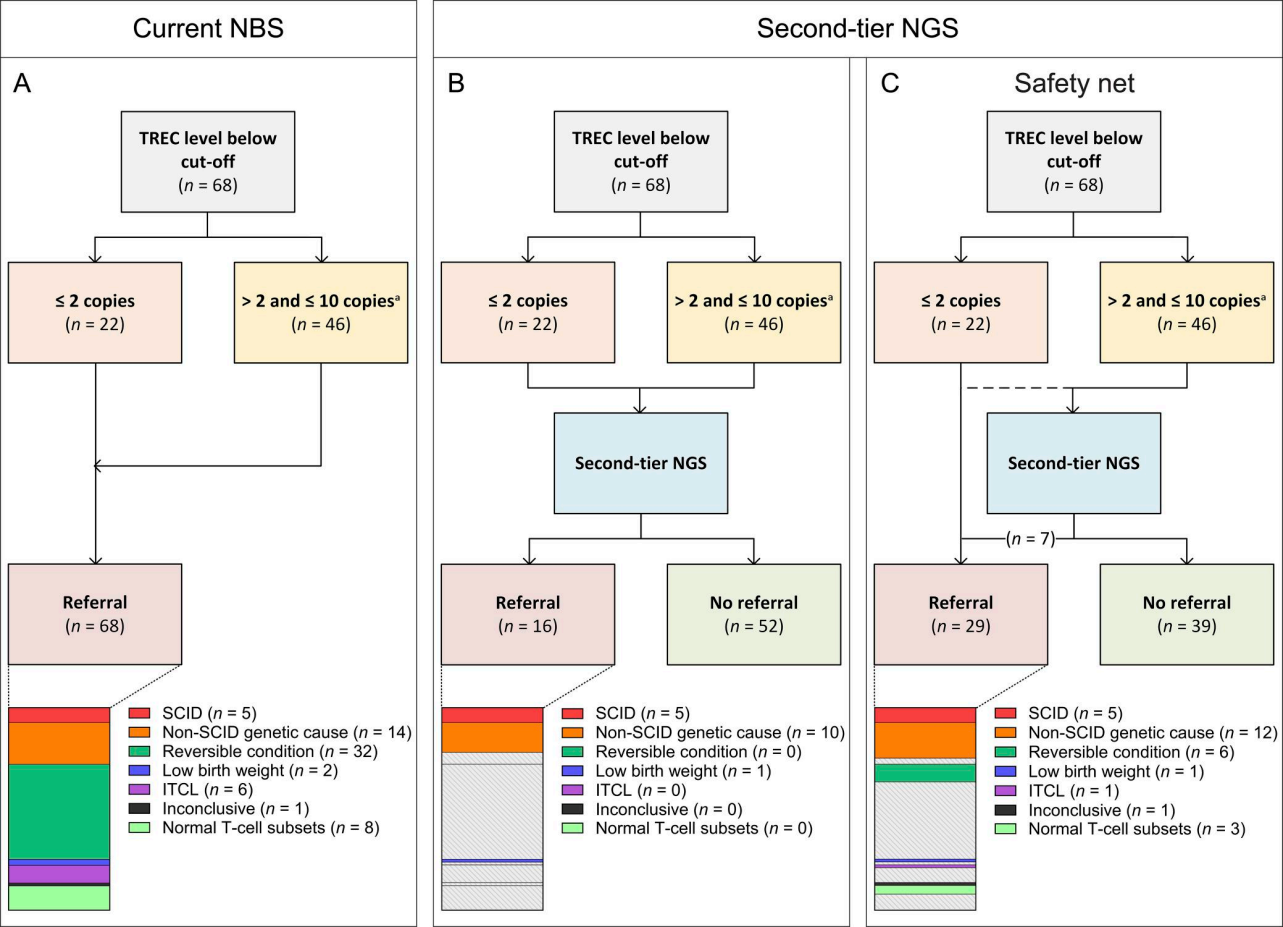
**Effect of second-tier NGS approaches on referrals. (A)** Diagnostic outcomes of the analyzed low-TREC cases (*n* = 68, ≤10 copies/3.2 mm punch, ImmunoIVD) following the current screening algorithm with first-tier TREC analysis only. A more detailed overview of the clinical characteristics is provided in Table S5. **(B and C)** The number of referred cases if second-tier NGS would be performed without a safety net (B) and with a safety net (C) to maintain high sensitivity for detecting SCID. With the safety net, second-tier NGS would be performed for all newborns with TRECs ≤10, but newborns with TRECs ≤2 would be referred directly after the first-tier result, with NGS results available after the initial clinical evaluation. Newborns with TRECs >2 and ≤10 would be referred only if NGS results are positive. ^a^Five newborns with TRECs >2 and ≤10 were indirectly referred after repeat analysis on a second NBS card according to the adjusted referral scheme after national implementation (see Materials and methods).

Of the 68 included NBS cases referred due to low TRECs, 22 had TREC values ≤2, including all SCID cases (Fig. 3 A and Table 1). The 14 cases with a non-SCID genetic cause for T cell lymphopenia comprised 10 IEI cases and 4 cases with (other) genetic syndromes (Table S5). Performing second-tier NGS on all 68 low-TREC cases would decrease the referrals to 16 (23.5%) (Fig. 3 B). To maintain high sensitivity for SCID, a safety net approach was evaluated. With the safety net, second-tier NGS would be performed for all newborns with low TRECs, but those with TRECs ≤2 would be directly referred after the first-tier TREC result, with NGS results following the initial clinical evaluation. Newborns with TRECs >2 and ≤10 would be referred only if NGS results are positive. Second-tier NGS with the safety net would result in 29 (42.6%) referrals, including 22 directly after first-tier testing (Fig. 3 C).

### Clinical impact

NGS results were compared with the collected patient follow-up data to assess the clinical impact of reducing referrals with the safety net approach (Fig. 3 C and Table S5). From the newborns that would not be referred (*n* = 39) with second-tier NGS with the safety net, eight were originally included in immunological outpatient follow-up after referral from TREC-based NBS: two with low TRECs due to maternal immunosuppressive drug use (case 32 and 33), along with the remaining newborn with low birth weight (case 53) and patients with ITCL (*n* = 5). For those with low TRECs attributed to maternal immunosuppressant use and low birth weight, follow-up was initiated to monitor lymphocyte subsets. Immunophenotyping was normalized at the first follow-up measurement, to which clinical follow-up was discontinued within 1 to 4 mo. No prophylactic treatment was started, and no infections occurred. Out of the five ITCL patients that would not be referred, only one received prophylactic treatment and experienced (viral) infections (case 55). Antibacterial prophylaxis was administered for 2.2 years after referral, during which the patient experienced viral respiratory tract infections (24). There were no complications, no hospital admissions, and the patient recovered from these infections quickly without treatment. After 2 years until the end of the evaluated follow-up period of ∼5 years, no infections occurred, and prophylactic treatment was not resumed. The two cases with a non-SCID genetic cause for T cell lymphopenia that would not be referred were patients with trisomy 21: one was not included in immunological follow-up, and the other was a critically ill newborn who died at 1 mo of age.

### Second-tier NGS increases PPV

Based on this cohort of 68 low-TREC cases, the PPV of the current screening algorithm with first-tier TREC analysis only would be 22.1%, with 15 patients diagnosed with SCID or another IEI, and the remaining cases classified as false-positive referrals (Table 2). Application of second-tier NGS identified one additional case with a heterozygous *TBX1* deletion (Table 1). Implementation of second-tier NGS with a safety net approach would have reduced referrals by 57.4% and increased the PPV to 55.2%. When limiting the screening target to SCID and thus only considering pathogenic or likely pathogenic variants in SCID-associated genes as true positives (25), the PPV would increase from 7.4% to 100% without the safety net and to 22.7% with the safety net (Table S6).

**Table 2.** Effect of second-tier NGS approaches on the PPV for SCID and other T cell deficiencies with a genetic cause

​	First-tier TREC results^a^, *n*		Referrals, *n*			Performance, %	
	≤2	>2 and ≤10^b^	Total	Genetic findings	False-positive	Reduction in referrals	PPV
**Current NBS**	22	46	68	15	53	NA	22.1%
**Second-tier NGS**							
Without safety net^c^	22	46	16	16	0	76.5%	100%^e^
With safety net^d^	22	46	29	16	13	57.4%	55.2%^e^

NA, not applicable.

a Copies/3.2 mm DBS punch (ImmunoIVD).

b Five newborns with TRECs >2 and ≤10 were indirectly referred after repeat analysis on a second NBS card according to the adjusted referral schema after national implementation (see Materials and methods).

c Second-tier NGS algorithm without a safety net, where only newborns with positive second-tier NGS results would be referred (Fig. 3 B).

d Second-tier NGS algorithm with a safety net, where newborns with TRECs ≤2 would be referred directly, and those with TRECs >2 and ≤10 only if NGS results are positive (Fig. 3 C).

e Considering all identified cases true-positive, although one new case was not (yet) diagnostically confirmed.

## Discussion

In this study, we demonstrated the effect of second-tier targeted NGS in TREC-based NBS on the PPV for SCID and other T cell deficiencies with a genetic cause. We included 68 newborns referred from NBS due to low TREC levels and retrospectively performed targeted amplicon sequencing of 105 genes associated with low TRECs and T cells at birth, according to the 2022 International Union of Immunological Sciences (IUIS) classification (25, 26). Using this second-tier approach, all SCID patients (*n* = 5) and 11 additional IEI patients were identified, increasing the PPV from 22.1% to 100%. However, pathogenic variants, such as deep-intronic or structural variants, may be missed by targeted NGS or may be located in novel or uncharacterized genes not included in the panel (27, 28). To maintain high sensitivity while integrating second-tier NGS, a safety net algorithm can be employed, where newborns with TRECs ≤2 would be referred directly, and those with TRECs >2 and ≤10 only if NGS results are positive. Applying this algorithm still showed a substantial reduction in referrals of 57.4% (*n* = 39), thereby increasing the PPV from 22.1% to 55.2%.

Due to the design of this retrospective study, we were able to combine our findings with patient follow-up data, providing unique insight into the clinical impact of reducing referrals from TREC-based NBS through second-tier NGS. Potentially actionable findings that would be missed by second-tier NGS are patients with ITCL in whom no genetic defect or underlying clinical cause for the low TRECs is found. Currently, these patients are included in clinical follow-up after referral, during which T cell lymphopenia may be transient or persistent (12, 29, 30). Although published long-term follow-up data are limited (24, 31), the clinical phenotype of ITCL patients identified through TREC-based NBS without severely decreased T cells appears to be mild without the occurrence of serious infections (24). As a result of second-tier NGS with the safety net algorithm, five out of the six included ITCL patients would not have been referred. These patients exhibited either no recurrent or severe infections or only a mild phenotype restricted to viral infections. Consequently, the clinical impact of not detecting ITCL patients without severely decreased TRECs or T cells by second-tier NGS seems acceptable and may even be considered beneficial from a parental perspective by preventing anxiety related to an ITCL diagnosis in their newborn. For ITCL patients with severely decreased T cells, or SCID patients with unknown genotypes, the safety net algorithm is designed to detect them regardless.

For this targeted NGS approach, a broad gene panel was designed that included not only SCID genes, but all genes linked to the phenotype of low TRECs and T cells at birth (*n* = 105) (25, 26). Discussions on the composition of NBS gene panels and subsequent reporting strategy, however, should remain ongoing and be guided by a multidisciplinary approach. Particular attention should be given to genes for which inclusion is debatable, such as *ATM*, which is associated with ataxia telangiectasia, a severe neurodegenerative disorder without curative treatment and a variable immunological phenotype (32, 33, 34). Including such genes in a second-tier panel requires more extensive parental counselling before NBS participation. Additionally, the integration of second-tier NGS into the screening program may necessitate re-evaluation of current informed consent procedures. In the Netherlands, information provision to parents about NBS begins during pregnancy and primarily focuses on its aim, procedure, and included conditions (35). Details on test methodologies are generally not provided, including DNA-sequencing methods already used in follow-up tier testing for cystic fibrosis and X-linked (XL) adrenoleukodystrophy. It remains to be determined whether information provision should be expanded to include test details when second-tier targeted NGS is implemented.

Targeted sequencing of a gene panel has both its advantages and disadvantages compared to whole-exome sequencing (WES) and whole-genome sequencing (WGS). By sequencing only genes of interest, targeted NGS aligns more appropriately with the principles of NBS than sequencing the entire exome or genome with, respectively, WES or WGS (36). As sequencing of a gene panel restricts genomic data generation to predefined targets, ethical concerns, including those related to public acceptability and parental uptake, are reduced (37, 38, 39). Additionally, data storage is smaller, which offers practical benefits, such as lower cost and improved sustainability. Disadvantages of a targeted NGS approach mostly lie with technical constraints. Sequencing of a gene panel is not as flexible as compared to WES or WGS, as the panel needs to be redesigned and revalidated whenever changes are implemented (28). With both gene panel sequencing and WES, deep-intronic variants are missed, and CNVs or other structural variants are either not detected or with lower accuracy (27, 28, 40). In our study, however, we were able to confirm a homozygous deletion of exons 1–3 in the *DCLRE1C* gene in one of the additional eight included DBS samples from SCID patients, as well as heterozygous *TBX1* deletions in the low-TREC cases referred from NBS with 22q11.2 deletion syndrome. Although all IEI patients with CNVs were identified in our cohort, it should be emphasized that amplicon sequencing is not a highly accurate method for CNV detection, particularly for smaller heterozygous CNVs. For those, alternative methods are recommended, such as a single nucleotide polymorphism array, multiplex ligation-dependent probe amplification, or qPCR (22, 41).

Besides increasing the PPV by reducing referrals, second-tier NGS also enables rapid identification of patients with a genetic variant in genes associated with SCID and other T cell deficiencies (23). In the Netherlands, when SCID is suspected after referral, urgent WES with SCID gene panel analysis is often requested, with results expected within 3 wk. NGS integrated in NBS as a second-tier test, therefore, has the potential to decrease the time until a genetic diagnosis, although this will depend on the frequency of sequencing. Moreover, it should be further investigated whether physicians would consider NGS results from NBS, together with nongenetic confirmatory tests such as immunophenotyping, sufficient to initiate treatment, or whether diagnostic genetic confirmation would remain required. Ideally, second-tier testing should be as frequent as possible to minimize the referral time, while remaining cost-effective. The cost-effectiveness will increase when NGS tests in follow-up tiers for other conditions are combined, for example, in NBS for inherited metabolic disorders, cystic fibrosis, or XL agammaglobulinemia and other B cell deficiencies (23, 42, 43, 44, 45).

Conducted within a screening laboratory, this study provided initial insight into the practical feasibility of second-tier NGS, which is essential for progressing toward integration into the screening program and highlights key considerations for implementation. Based on logged experiences, the turnaround time when handling a smaller number of samples in a second-tier setting was estimated to be 4 days. With our safety net algorithm, second-tier NGS could replace the second NBS card that is currently requested in the Dutch screening program for preterm newborns and newborns with TRECs >2 and ≤10. In newborns with initial TRECs >2 and ≤10, repeat TREC analysis on a second NBS card has been shown to effectively reduce the referral rate from 0.03% to, on average, 0.01% (20, 46), but does increase the referral time until initial clinical evaluation to up to 3 wk after birth, instead of ∼1 to 2 wk. Second-tier NGS would likely reduce the referral time by eliminating the need for a second NBS card, which also reduces the emotional burden on parents. Further adaptations to the screening algorithm could be considered, such as increasing the TREC cutoff for second-tier testing, similar to the approach applied in Norway, where second-tier NGS is implemented in the screening program (23). This may allow the detection of leaky SCID and other T cell deficiencies with TRECs >10 that would otherwise be missed. Before implementation into NBS, a prospective evaluation in parallel to the current screening program will be needed to evaluate real-time feasibility and robustness, cost-effectiveness, and optimization of the algorithm.

### Conclusion

This study demonstrated the potency of second-tier NGS to improve screening accuracy for SCID and other T cell deficiencies with a genetic cause. By directly referring newborns with profoundly reduced TRECs using a safety net algorithm, a high sensitivity for detecting SCID could be maintained while substantially increasing the PPV. No patients with severe immunological phenotypes were missed, suggesting that not detecting non-severe T cell lymphopenia patients without a genetic diagnosis appears clinically acceptable.

## Materials and methods

### Dutch screening algorithm

TREC quantification is conducted with the SPOT-it TREC & SMN1 Screening Kit (ImmunoIVD) using β-actin (*ACTB*) as a reference gene, with a cutoff of ≤10 TREC copies/3.2 mm DBS punch. During the pilot study (April 2018–December 2020), all newborns with TRECs below the cutoff were referred to a pediatric immunologist. After national implementation in January 2021, the screening algorithm was adjusted to reduce referrals: newborns with TRECs ≤2 copies/punch are directly referred, whereas for newborns with TRECs >2 and ≤10 copies/punch, a second NBS card is requested after 7 days. Referral occurs only if TRECs then remain below the cutoff of ≤10 copies/punch (indirect referral). For preterm newborns with abnormal TRECs, a second card is also requested after a gestational age of 36 wk. A detailed overview of the Dutch screening algorithm was previously published (20, 24).

### Sample collection

We included NBS cards from 68 newborns referred with low TRECs, comprising 54 newborns from the pilot study and 14 newborns referred after national implementation, for whom informed consent was obtained. Clinical data from these referrals were collected at the Dutch academic medical centers and managed using Castor EDC (v2024.2.1.0). Detailed data have been previously published (24). Additionally, NBS cards of 100 anonymous healthy newborns with normal TRECs, analyzed between 2021 and 2023, were included. Before collection, NBS cards were stored at the National Institute for Public Health and the Environment at −20°C. The use of stored NBS cards was approved by the Neonatal Screening Research Workgroup (nr. 2023-05). Additional DBS cards from eight SCID patients stored at −20°C in the Willem Alexander Children’s Hospital BioBank were included with different affected genes: three in *RAG1*, two in *RAG2*, one in *IL2RG*, one in *NHEJ1*, and one in *DCLRE1C*. These DBS cards were manufactured in 2013 from peripheral EDTA blood samples. The study was reviewed and approved by the Institutional Review Board of the Leiden University Medical Center (nr. 23-3127 and nr. 23-3060).

### Gene panel design

Gene selection was performed based on the 2022 IUIS classification for human IEIs (25). Genes were selected when associated with a phenotype of low TRECs and T cell lymphopenia at birth (26). The custom AmpliSeq (Illumina) gene panel consisted of 105 genes covered by 3,003 amplicons (Table S7). Primers were designed to provide amplicons with an average of 261 bp with 25 bp exon padding and a predicted coverage of 99.44%.

### NGS workflow

DNA was extracted from one 3.2 mm DBS punch following the two-step lysis protocol (number five) described by Duintjer et al. (2025) (47, 48). DNA concentration was measured with the Qubit^tm^ 1X dsDNA High Sensitivity Assay Kit on the Invitrogen Qubit^tm^ Fluorometer (Thermo Fisher Scientific). Libraries were prepared following the AmpliSeq^tm^ for Illumina protocol with two primer pools. A volume of 7.5 µl of undiluted DNA was used to maximize input. Library quantity and fragment size were assessed with the D5000 High Sensitivity ScreenTape Assay on the 4200 TapeStation System (Agilent Technologies). Libraries were then diluted to a starting concentration of 2 nM, pooled, and spiked with 5% PhiX Control V3 (Illumina). Sequencing was performed on the NextSeq2000tm system with the NextSeq™ 1000/2000 P2 XLEAP-SBS™ Reagent Kit (300 Cycles; Illumina). For samples that were repeated, sequencing was performed on the iSeq 100^tm^ System (Illumina). BCL files were demultiplexed and converted to FASTQ files by an in-house pipeline using Illumina bcl2fastq2 Conversion Software v2.20.0.422 (Linux rpm). Secondary data analysis was performed using the Illumina DRAGEN amplicon pipeline (v4.3.6), aligning reads to the GRCh38 reference genome. For CNV analysis, a Panel of Normals was created from 92 of the NBS cases with normal TRECs.

### Variant filtering and reporting strategy

Variant prioritization and interpretation were performed using Emedgene software (Illumina, version 37.5.2). Variants meeting one of the following filter criteria were prioritized: (1) pathogenic or likely pathogenic variants reported in ClinVar with an allele frequency (AF) of ≤0.5 in the Genome Aggregation Database to reduce submission errors in ClinVar and (2) truncating and frameshift variants not previously reported as pathogenic or likely pathogenic in ClinVar with an AF of ≤0.001 (Fig. 2 A). Additional variants (3) with an AF of ≤0.0005 were filtered to detect VUSs in cases where only one pathogenic or likely pathogenic variant was found in a gene with AR inheritance to increase sensitivity. Finally, the Emedgene AI shortlist preset was assessed to identify additional relevant variants not captured by the other filters. Prioritized variants were selected for manual review, except when only a single heterozygous variant was filtered in an AR gene. A detailed overview of the applied filters is provided in Table S2.

Variant interpretation was performed following the American College of Medical Genetics and Genomics guidelines (49) by two independent reviewers, blinded to the original sample names and NBS outcomes. Assessed variant databases included ClinVar, the Leiden Open Variation Database, and the Dutch Society for Laboratory Specialists Clinical Genetics database. Pathogenic or likely pathogenic variants were reported when present heterozygous in genes with autosomal dominant (AD) inheritance, hemizygous and homozygous in genes with XL inheritance, and bi-allelic in genes with AR inheritance (Fig. 2 B). Heterozygous pathogenic or likely pathogenic variants in genes with AR and XL inheritance were only reported when an additional VUS was identified. After review, results were integrated with the NBS outcomes and clinical data to confirm findings.

### Online supplemental material

The supplemental material includes the percentage of samples with sufficient read depth per amplicon (Fig. S1), the reviewed variants classified as VUS (Table S1), the variant filter settings applied in Emedgene (Table S2), the reviewed variants classified as (likely) pathogenic in the positive NBS cases (Table S3), the additionally included SCID cases (Table S4), the clinical characteristics of low-TREC NBS cases (Table S5), the effect of second-tier NGS approaches on the PPV for SCID (Table S6), and the second-tier gene panel (Table S7).

## Supplementary Material

Table S1 shows reviewed variants classified as VUS.

Table S2 shows variant filter settings applied in Emedgene.

Table S3 shows reviewed variants classified as (likely) pathogenic in the positive NBS cases.

Table S4 shows additionally included SCID cases.

Table S5 shows clinical characteristics of low-TREC NBS cases (*n* = 68).

Table S6 shows effect of second-tier NGS approaches on the PPV for SCID.

Table S7 shows second-tier gene panel (*n* total = 105).

## Data Availability

All data generated or analyzed during this study that are not included in the article or online supplemental material are available from the corresponding author upon reasonable request.
