# Supplementary material for: Second-tier genetics improves newborn screening accuracy for SCID and other T cell deficiencies
Source: J Hum Immun. 2026 Jul 16;2(5):e20260031. doi: 10.70962/jhi.20260031 (PMC13374527; doi:10.70962/jhi.20260031)
Supplement: Table S1 — shows reviewed variants classified as VUS. [file jhi_20260031_tables1.docx]

**Table S1.** Reviewed variants classified as VUS

| **VUS No.** | **Gene (MOI)** | **Variant** | **In silico prediction** | **Allele frequency^a^** | **Variant databases** | **ACMG criteria^b^** |
| --- | --- | --- | --- | --- | --- | --- |
| 1 | *GFI1* (AD) | NM_005263.5: c.1091-4A>T | SpliceAI: 0 | NR | NR | PM2, BP4 |
| 2 | *CDC42* (AD) | NM_044472.3: c.487-4G>C | SpliceAI: 0.06 | 0.000001592 | NR | PM2, BP4 |
| 3 | *STAT3* GOF (AD) | NM_139276.3: c.82A>G  p.(Met28Val) | SpliceAI: 0  REVEL: 0.435 | 0.000001591 | ClinVar: VUS (1x), LP (1x) | PM2, PP2, BP4 |
| 4 | *STAT1* LOF (AD) | NM_007315.4: c.1038-14A>C | SpliceAI: 0 | NR | NR | PM2, BP4 |
| 5 | *TBX1* (AD) | NM_080647.1: c.841-67C>T | SpliceAI: 0.24 | 0.000 | NR | PM2, PP3 |
| 6 | *FOXN1* (AD) | NM_003593.3: c.428C>A  p.(Ala143Asp) | SpliceAI: 0  REVEL: 0.552 | 0.000001592 | NR | PM2, BP4 |
| 7 | *JAK3* (AR) | NM_000215.4: c.430G>A  p.(Asp144Asn) | SpliceAI: 0.08  REVEL: 0.685 | 0.000 | LOVD: VUS | PM2, PM5, PP3 |
| 8 | *STAT1* LOF (AD) | NM_007315.4: c.129-21T>A | SpliceAI: 0.02 | 0.0002549 | NR | BS1 |
| 9 | *DKC1* (XL) | NM_001363.5: c.1477-9T>G | SpliceAI: 0.01 | NR | NR | PM2, BP4 |
| 10 | *SOCS1* (AD) | NM_003745.2: c.145G>T  p.(Ala49Ser) | SpliceAI: 0  REVEL: 0.048 | 0.0001058 | ClinVar: VUS | BP4, BS1 |
| 11 | *BACH2* (AD) | NM_021813.4: c.1427G>T  p.(Ser476Ile) | SpliceAI: 0.01  REVEL: 0.101 | 0.00004225 | ClinVar: VUS | PM2, BP4 |
| 12 | *TBX1* (AD) | NM_080647.1: c.70G>T  p.(Ala24Ser) | SpliceAI: 0.01  REVEL: 0.206 | 0.00002226 | ClinVar: VUS | PM2, BP4 |
| 13 | *FOXN1* (AD) | NM_003593.3: c.905C>T  p.(Thr302Met) | SpliceAI: 0  REVEL: 0.529 | 0.00006146 | ClinVar/LOVD: VUS | BS1, BP4 |
| 14 | *CHD7* (AD) | NM_017780.4: c.1708A>G  p.(Met570Val) | SpliceAI: 0  REVEL: 0.102 | 0.00002746 | ClinVar: B, LOVD: VUS | BP4 |
| 15 | *MSN* (XL) | NM_002444.3: c.167C>G  p.(Ser56Cys) | SpliceAI: 0  REVEL: 0.392 | NR | NR | PM2, BP4 |
| 16 | *FOXN1* (AD) | NM_003593.3: c.1877C>T  p.(Thr626Met) | SpliceAI: 0  REVEL: 0.265 | 0.00005126 | ClinVar: VUS | BS1, BP4 |
| 17 | *GATA2* (AD) | NM_032638.5: c.224C>T  p.(Ala75Val) | SpliceAI: 0  REVEL: 0.672 | 0.000002598 | ClinVar: VUS | PM2, PP2, PP3 |
| 18 | *FOXN1* (AD) | NM_003593.3: c.1454C>T  p.(Pro485Leu) | SpliceAI: 0.03  REVEL: 0.485 | 0.00006662 | ClinVar/LOVD: VUS | BS1, BP4 |
| 19 | *CHD7* (AD) | NM_017780.4: c.8168G>C  p.(Ser2723Thr) | SpliceAI: 0  REVEL: 0.147 | 0.000002571 | NR | PM2, BP4 |
| 20 | *GATA2* (AD) | NM_032638.5: c.707T>C  p.(Met236Thr) | SpliceAI: 0  REVEL: 0.603 | 0.00003074 | ClinVar: VUS, LOVD: LB | PM2, PP2, BP4 |
| 21 | *SOCS1* (AD) | NM_003745.2: c.144_149del  p.(Ala49_Pro50del) | SpliceAI: 0 | 0.0002803 | ClinVar/LOVD/VKGL: VUS | PM4, BS1 |
| 22 | *STAT1* LOF (AD) | NM_007315.4: c.1341C>A  p.(Asp447Glu) | SpliceAI: 0  REVEL: 0.289 | 0.0001805 | ClinVar: VUS (3x), LB (1x) | PP2, BS1, BP4 |

ACMG, American College of Medical Genetics and Genomics; AD, autosomal dominant; AR, autosomal recessive; B, benign; GOF, gain of function; LB, likely benign; LOF, loss of function; LOVD, Leiden Open Variation Database; LP, likely pathogenic; MOI, mode of inheritance; NR, not reported; VKGL, Dutch Society for Laboratory Specialists Clinical Genetics; VUS, variant of uncertain significance; XL, X-linked.

^a^ According to the total allele frequency reported in GnomAD.

^b^ Assigned using Emedgene software (Illumina, version 37.5.2). Variant interpretation was performed according to the ACMG guidelines (S1) with pathogenic criteria weighted as very strong (PVS1), strong (PS1-4), moderate (PM1-6), or supporting (PP1-5), and benign criteria weighted as stand-alone (BA1), strong (BS1-4), or supporting (BP1-6).

48. Richards S, Aziz N, Bale S, Bick D, Das S, Gastier-Foster J, et al. Standards and guidelines for the interpretation of sequence variants: a joint consensus recommendation of the American College of Medical Genetics and Genomics and the Association for Molecular Pathology. Genet Med. 2015;17(5):405-24. doi: 10.1038/gim.2015.30.
