# Supplementary material for: Second-tier genetics improves newborn screening accuracy for SCID and other T cell deficiencies
Source: J Hum Immun. 2026 Jul 16;2(5):e20260031. doi: 10.70962/jhi.20260031 (PMC13374527; doi:10.70962/jhi.20260031)
Supplement: Table S2 — shows variant filter settings applied in Emedgene. [file jhi_20260031_tables2.docx]

**Table S2.** Variant filter settings applied in Emedgene

| **Filter preset** | | **Quality filters** | **Polymorphism filters^a^** | **Variant effect filters (S2, S3)** |
| --- | --- | --- | --- | --- |
| 1 | ClinVar P/LP variants | None | AF ≤ 0.5 | Known pathogenic variants true |
| 2 | High effect variants | Allele bias ≥ 20  Depth ≥ 20  Mapping quality ≥ 30  VCF filter pass | AF ≤ 0.001 | Splice acceptor/donor variant, stop gained, frameshift variant, stop/start lost, predicted_splice_high, predicted_splice_moderate |
| 3 | VUS strategy |  | AF ≤ 0.0005 | Inframe insertion/deletion, missense variant, protein altering variant, splice donor region variant, splice polypyrimidine tract variant, splice region variant, splice donor 5th base variant, splice_region_high_conservation |

AF, allele frequency; LP, likely pathogenic; P, pathogenic; VCF, variant call format; VUS, variant of uncertain significance.

^a^ According to the total allele frequency reported in GnomAD.

49. Eilbeck K, Lewis SE, Mungall CJ, Yandell M, Stein L, Durbin R, et al. The Sequence Ontology: a tool for the unification of genome annotations. Genome Biol 2005; 6:R44. doi: 10.1186/gb-2005-6-5-r44.

50. McLaren W, Gil L, Hunt SE, Riat HS, Ritchie GR, Thormann A, et al. The Ensembl Variant Effect Predictor. Genome Biol 2016; 17:122. doi: 10.1186/s13059-016-0974-4.
