# Supplementary material for: Second-tier genetics improves newborn screening accuracy for SCID and other T cell deficiencies
Source: J Hum Immun. 2026 Jul 16;2(5):e20260031. doi: 10.70962/jhi.20260031 (PMC13374527; doi:10.70962/jhi.20260031)
Supplement: Table S3 — shows reviewed variants classified as (likely) pathogenic in the positive NBS cases. [file jhi_20260031_tables3.docx]

**Table S3.** Reviewed variants classified as (likely) pathogenic in the positive NBS cases

| **Case No.** | **Gene (MOI)** | **Variant** | **In silico prediction** | **Allele frequency^a^** | **Variant databases** | **ACMG criteria^b^** |
| --- | --- | --- | --- | --- | --- | --- |
| 1 | *RAG1* (AR) | NM_000448.3: c.519del  p.(Glu174SerfsTer27) | SpliceAI: 0 | 0.00001272 | ClinVar: P | PVS1, PS3, PM2 |
| 2 | *RAG1* (AR) | NM_000448.3: c.1331C>T  p.(Ala444Val) | SpliceAI: 0  REVEL: 0.6 | 0.00001281 | ClinVar/LOVD: P, VKGL: (L)P | PS3, PM1, PM2, PM5, PP2, BP4 |
| 3 | *RAG1* (AR) | NM_000448.3: c.2095C>T  p.(Arg699Trp) | SpliceAI: 0  REVEL: 0.817 | 0.000007686 | ClinVar/VKGL: (L)P, LOVD: P | PS3, PM1, PM2, PM5, PP2, PP3 |
| 3 | *RAG1* (AR) | NM_000448.3:  c.2974A>G p.(Lys992Glu) | SpliceAI: 0  REVEL: 0.595 | 0.00003073 | ClinVar/LOVD: P, VKGL (L)P | PS3, PM1, PM2, PM5, PP2, BP4 |
| 4 | *IL2RG* (XL) | NM_000206.3: c.298C>T  p.(Gln100Ter) | SpliceAI: 0 | NR | LOVD: P, VKGL: (L)P | PVS1, PM2, PP5 |
| 5 | *IL2RG* (XL) | NM_000206.3: c.190G>A  p.(Val64Met) | SpliceAI: 0  REVEL: 0.645 | NR | ClinVar: VUS, LOVD: P | PM1, PM2, PP2, PP3 |
| 6 | *FOXN1* (AD/AR) | NM_003593.3: c.831-2A>G  p.(?) | SpliceAI: 1 | NR | LOVD: LP | PVS1, PM2, PP5 |
| 7 | *FOXN1* (AD/AR) | NM_003593.3: c.143del  p.(Cys48SerfsTer254) | SpliceAI: 0 | NR | LOVD: P | PVS1, PM2, PP5 |
| 8 | *FOXN1* (AD/AR) | NM_003593.3: c.1079T>C  p.(Leu360Pro) | SpliceAI: 0  REVEL: 0.883 | NR | LOVD/VKGL: VUS | PM1, PM2, PM6, PP3 |
| 9^c^ | *RMRP* (AR) | NR_003051.3: n.147G>A | NR | 0.00003712 | ClinVar/LOVD/VKGL: (L)P | PS3, PM2, PP5 |
| 9^c^ | *RMRP* (AR) | NR_003051.3: n.-32_1dup | NR | NR | VKGL: (L)P | PM2, PP5 |
| 10 | *ATM* (AR) | NM_000051.4: c.5979_5983del  p.(Ser1993ArgfsTer23) | NR | 0.000003185 | ClinVar: P, VKGL: (L)P | PVS1, PS3, PM2 |
| 10 | *ATM* (AR) | NM_000051.4: c.7875_7876delinsGC  p.(Asp2625_Ala2626delinsGluPro) | NR  ACMG, American College of Medical Genetics and Genomics; AD, autosomal dominant; AR, autosomal recessive; LOVD, Leiden Open Variation Database; LP, likely pathogenic; MOI, mode of inheritance; NR, not reported; P, pathogenic; VKGL, Dutch Society for Laboratory Specialists Clinical Genetics; VUS, variant of uncertain significance; XL, X-linked.  ^a^ According to the total allele frequency reported in GnomAD.  ^b^ Assigned using Emedgene software (Illumina, version 37.5.2). Variant interpretation was performed according to the ACMG guidelines (S1) with pathogenic criteria weighted as very strong (PVS1), strong (PS1-4), moderate (PM1-6), or supporting (PP1-5), and benign criteria weighted as stand-alone (BA1), strong (BS1-4), or supporting (BP1-6).  ^c^ Variant in RNA-coding gene, for which classification was largely based on (diagnostic) variant databases. | NR | ClinVar/VKGL: (L)P, LOVD: P | PS3, PM2, PP5 |
