# Supplementary material for: Second-tier genetics improves newborn screening accuracy for SCID and other T cell deficiencies
Source: J Hum Immun. 2026 Jul 16;2(5):e20260031. doi: 10.70962/jhi.20260031 (PMC13374527; doi:10.70962/jhi.20260031)
Supplement: Table S4 — shows additionally included SCID cases. [file jhi_20260031_tables4.docx]

**Table S4**. Additionally included SCID cases

| **Case ID** | **Gene (MOI)** | **Variant** | **Zygosity** |
| --- | --- | --- | --- |
| DBS-1 | *RAG1* (AR) | NM_000448.3: c.433del  p.(Glu145LysfsTer19) and  c.2326C>T  p.(Arg776Trp) | Compound heterozygous |
| DBS-2 | *RAG1* (AR) | NM_000448.3: c.1767C>G  p.(Tyr589Ter) | Homozygous |
| DBS-3 | *RAG1* (AR) | NM_000448.3 c.1180C>T  p.(Arg394Trp) | Homozygous |
| DBS-4 | *RAG2* (AR) | NM_000536.4: c.95G>A  p.(Gly32Glu) and  c.1352G>C  p.(Gly451Ala) | Compound heterozygous |
| DBS-5 | *RAG2* (AR) | NM_000536.4: c.104G>C  p.(Gly35Ala) and *missed*  *c.413A>G*  *p.(Tyr138Cys)* | Compound heterozygous |
| DBS-6 | *IL2RG* (XL) | NM_000206.3: c.3G>T  p.(Met1?) | Hemizygous |
| DBS-7 | *NHEJ1* (AR) | NM_024782.3: c.532C>T  p.(Arg178Ter) | Homozygous |
| DBS-8 | *DCLRE1C* (AR) | NM_001033855.3: deletion exons 1-3 | Homozygous |

AR, autosomal recessive; DBS, dried blood spot; MOI, mode of inheritance; SCID, severe combined immunodeficiency; XL, X-linked.

Additional DBS cards from eight SCID patients, stored in the Willem Alexander Children’s Hospital biobank of the Leiden University Medical Center, were analyzed. These DBS cards were manufactured in 2013 from peripheral EDTA blood samples and therefore did not include material from the screening program.
