# Supplementary material for: Second-tier genetics improves newborn screening accuracy for SCID and other T cell deficiencies
Source: J Hum Immun. 2026 Jul 16;2(5):e20260031. doi: 10.70962/jhi.20260031 (PMC13374527; doi:10.70962/jhi.20260031)
Supplement: Table S5 — shows clinical characteristics of low-TREC NBS cases (n = 68). [file jhi_20260031_tables5.docx]

**Table S5**. Clinical characteristics of low-TREC NBS cases (*n* = 68)

| **Case no.** | **Diagnosis** | **Referral** | | **Clinical characteristics** | | | |
| --- | --- | --- | --- | --- | --- | --- | --- |
|  |  | **Without safety net** | **With safety net** | **HSCT/gene therapy** | **Follow-up^a^** | **Prophylactic treatment** | **Infections** |
| **SCID** | | | | | | | |
| 1 | *RAG1* | + | + | + | + | + | - |
| 2 | *RAG1* | + | + | + | + | + | - |
| 3 | *RAG1* | + | + | + | + | + | - |
| 4 | *IL2RG* | + | + | + | + | + | - |
| 5 | *IL2RG* | + | + | + | + | + | - |
| **Non-SCID with genetic cause** | | | | | | | |
| 6 | *FOXN1* haploinsufficiency | + | + | - | + | + | + |
| 7 | *FOXN1* haploinsufficiency | + | + | - | + | - | - |
| 8 | *FOXN1* haploinsufficiency | + | + | - | + | - | - |
| 9 | Cartilage hair hypoplasia | + | + | + | + | + | - |
| 10 | Ataxia telangiectasia | + | + | - | + | + | - |
| 11 | 22q11.2 deletion syndrome | + | + | - | + | + | + |
| 12 | 22q11.2 deletion syndrome | + | + | - | + | - | - |
| 13 | 22q11.2 deletion syndrome | + | + | - | Deceased |  |  |
| 14 | 22q11.2 deletion syndrome | + | + | - | + | - | - |
| 15 | 22q11.2 deletion syndrome | + | + | - | + | - | - |
| 16 | Trisomy 21 | - | - | - | Deceased |  |  |
| 17 | Trisomy 21 | - | - | - | - |  |  |
| 18 | Noonan syndrome | - | + | - | + | - | - |
| 19 | Rothmund-Thomson syndrome | - | + | + | Deceased |  |  |
| **Reversible conditions with T cell impairment** | | | | | | | |
| 20 | Chylothorax/  hydrops | - | + | - | Deceased |  |  |
| 21 | Chylothorax/  hydrops | - | - | - | Deceased |  |  |
| 22 | Chylothorax/  hydrops | - | + | - | - |  |  |
| 23 | Chylothorax/  hydrops | - | - | - | - |  |  |
| 24 | Chylothorax/  hydrops | - | + | - | - |  |  |
| 25 | Chylothorax/  hydrops | - | - | - | Deceased |  |  |
| 26 | Infection/sepsis | - | - | - | - |  |  |
| 27 | Infection/sepsis | - | + | - | - |  |  |
| 28 | Infection/sepsis | - | - | - | - |  |  |
| 29 | Infection/sepsis | - | - | - | - |  |  |
| 30 | Infection/sepsis | - | - | - | - |  |  |
| 31 | Infection/sepsis | - | - | - | - |  |  |
| 32 | Maternal immunosuppressants | - | - | - | + | - | - |
| 33 | Maternal immunosuppressants | - | - | - | + | - | - |
| 34 | Maternal immunosuppressants | - | + | - | + | + | - |
| 35 | Maternal immunosuppressants | - | + | - | + | + | - |
| 36 | Cardiac anomaly | - | - | - | - |  |  |
| 37 | Cardiac anomaly | - | - | - | - |  |  |
| 38 | Cardiac anomaly | - | - | - | - |  |  |
| 39 | Multimorbidity | - | - | - | - |  |  |
| 40 | Multimorbidity | - | - | - | - |  |  |
| 41 | Multimorbidity | - | - | - | - |  |  |
| 42 | Multimorbidity | - | - | - | - |  |  |
| 43 | Multimorbidity | - | - | - | - |  |  |
| 44 | Multimorbidity | - | - | - | Deceased |  |  |
| 45 | Multimorbidity | - | - | - | - |  |  |
| 46 | Multimorbidity | - | - | - | Deceased |  |  |
| 47 | Congenital diaphragmatic hernia | - | - | - | - |  |  |
| 48 | Congenital diaphragmatic hernia | - | - | - | Deceased |  |  |
| 49 | Congenital diaphragmatic hernia | - | - | - | Deceased |  |  |
| 50 | Congenital diaphragmatic hernia | - | - |  | Deceased |  |  |
| 51 | Chemotherapy | - | - | - | - |  |  |
| **Low birth weight alone** | | | | | | | |
| 52 | Low birth weight | + | + | - | + | - | - |
| 53 | Low birth weight | - | - | - | + | - | - |
| **Idiopathic T cell lymphopenia (ITCL)** | | | | | | | |
| 54 | ITCL | - | + | - | + | + | - |
| 55 | ITCL | - | - | - | + | + | + |
| 56 | ITCL | - | - | - | + | - | - |
| 57 | ITCL | - | - | - | + | - | - |
| 58 | ITCL | - | - | - | + | - | - |
| 59 | ITCL | - | - | - | + | - | - |
| **Inconclusive** | | | | | | | |
| 60 | Lost to follow-up | - | + | - | + | - | - |
| **Normal T cell subsets without other cause for low TRECs** | | | | | | | |
| 61 | SNP in TREC region | - | - | - | - |  |  |
| 62 | SNP in TREC region | - | + | - | - |  |  |
| 63 | SNP in TREC region | - | + | - | - |  |  |
| 64 | SNP in TREC region | - | - | - | - |  |  |
| 65 | Unknown | - | - | - | - |  |  |
| 66 | Unknown | - | - | - | - |  |  |
| 67 | Unknown | - | + | - | - |  |  |
| 68 | Unknown | - | - | - | - |  |  |

HSCT, hematopoietic stem cell transplantation; ITCL, idiopathic T cell lymphopenia; SCID, severe combined immunodeficiency; SNP, single nucleotide polymorphism; TREC, T cell receptor excision circle.

Clinical characteristics (+, present; -, absent) of newborns with low TRECs that would be referred (+) or not be referred (-) with the two second-tier NGS approaches: without (Figure 3B) and with safety net (Figure 3C).

**^a^** Refers to immunological outpatient clinic follow-up.
