# Supplementary material for: Second-tier genetics improves newborn screening accuracy for SCID and other T cell deficiencies
Source: J Hum Immun. 2026 Jul 16;2(5):e20260031. doi: 10.70962/jhi.20260031 (PMC13374527; doi:10.70962/jhi.20260031)
Supplement: Table S6 — shows effect of second-tier NGS approaches on the PPV for SCID. [file jhi_20260031_tables6.docx]

**Table S6**. Effect of second-tier NGS approaches on the PPV for SCID

|  | **First-tier TREC results^a^, *n*** | | **Referrals, *n*** | | | | **Performance, %** | |
| --- | --- | --- | --- | --- | --- | --- | --- | --- |
|  | **≤ 2** | **> 2 and ≤ 10^b^** | **Total** | | **SCID** | **False-positive** | **Reduction in referrals** | **PPV** |
| **Current NBS** | 22 | 46 | 68 | | 5 | 63 | NA | 7.4% |
| **Second-tier NGS** | | | |  | | | | |
| Without safety net^c^ | 22 | 46 | 5 | | 5 | 0 | 92.6% | 100% |
| With safety net^d^ | 22 | 46 | 22 | | 5 | 17 | 67.6% | 22.7% |

IEI, inborn error of immunity; IUIS, International Union of Immunological Societies; NA, not applicable; NBS, newborn screening; NGS, next-generation sequencing; PPV, positive predictive value; SCID, severe combined immunodeficiency; TREC, T cell receptor excision circle.

Observed reductions in referrals and increases in PPV for second-tier NGS strategies with and without a safety net algorithm, when only pathogenic variants in SCID-associated genes would be considered true-positive results, and all other findings would be false-positive. According to the 2022 IUIS classification for human IEIs, these genes are: *ADA, AK2, CD3D, CD3E, CD3Z, CORO1A, DCLRE1C, IL2RG, IL7R, JAK3, LAT, LCP2, LIG4, NHEJ1, PRKDC, PTPRC, RAC2, RAG1,* and *RAG2* (S4).

^a^ Copies/3.2 mm DBS punch (ImmunoIVD).

^b^ Five newborns with TRECs > 2 and ≤ 10 were indirectly referred after repeat analysis on a second NBS card according to the adjusted referral schema after national implementation (see materials and methods).

^c^ Second-tier NGS algorithm without a safety net, where only newborns with positive second-tier NGS results would be referred (Figure 3B).

^d^ Second-tier NGS algorithm with a safety net, where newborns with TRECs ≤ 2 would be referred directly, and those with TRECs > 2 and ≤ 10 only if NGS results are positive (Figure 3C).

25. Tangye SG, Al-Herz W, Bousfiha A, Cunningham-Rundles C, Franco JL, Holland SM, et al. Human Inborn Errors of Immunity: 2022 Update on the Classification from the International Union of Immunological Societies Expert Committee. J Clin Immunol 2022; 42:1473-507. doi: 10.1007/s10875-022-01289-3
