# Supplementary material for: Second-tier genetics improves newborn screening accuracy for SCID and other T cell deficiencies
Source: J Hum Immun. 2026 Jul 16;2(5):e20260031. doi: 10.70962/jhi.20260031 (PMC13374527; doi:10.70962/jhi.20260031)
Supplement: Table S7 — shows second-tier gene panel (n total = 105). [file jhi_20260031_tables7.docx]

**Table S7.** Second-tier gene panel (*n* total = 105)

| **Gene** | **MOI** | **Phenotype** |
| --- | --- | --- |
| *ADA* | AR | ADA deficiency |
| *ADA2* | AR | ADA2 deficiency |
| *AK2* | AR | AK2 defect |
| *ARPC1B* | AR | Arp2/3-mediated filament branching defect |
| *ATM* | AR | Ataxia-telangiectasia |
| *B2M* | AR | MHC class I deficiency |
| *BACH2* | AD | BACH2 deficiency |
| *BCL11B* | AD | BCL11B deficiency |
| *CARD11* | AR LOF | CARD11 deficiency |
| *CCBE1* | AR | Hennekam-lymphangiectasialymphedema syndrome |
| *CD3D* | AR | CD3δ deficiency |
| *CD3E* | AR | CD3ε deficiency |
| *CD3G* | AR | CD3γ deficiency |
| *CD3Z* | AR | CD3ζ deficiency |
| *CD40LG* | XL | CD40 ligand (CD154) deficiency |
| *CD8A* | AR | CD8 deficiency |
| *CDC42* | AD | CDC42 deficiency |
| *CDCA7* | AR | Immunodeficiency with centromeric instability and facial anomalies (ICF type 3) |
| *CEBPE* | AR GOF | CEBPE neofunction |
| *CHD7* | AD | CHARGE syndrome |
| *CIITA* | AR | MHC class II deficiency group A |
| *COPG1* | AR | COPG1 deficiency |
| *CORO1A* | AR | Coronin-1A deficiency |
| *CRACR2A* | AR | CRACR2A deficiency |
| *CTLA-4* | AD | CTLA4 haploinsufficiency (ALPS-V) |
| *CTPS1* | AR | CTPS1 deficiency |
| *DCLRE1C* | AR | DCLRE1C (Artemis) deficiency |
| *DEF6* | AR | DEF6 deficiency |
| *DIAPH1* | AR | DIAPH1 deficiency |
| *DKC1* | XL | DKCX1 |
| *DNMT3B* | AR | Immunodeficiency with centromeric instability and facial anomalies (ICF type 1) |
| *DOCK2* | AR | DOCK2 deficiency |
| *DOCK8* | AR | DOCK8 deficiency |
| *ERCC6L2* | AR | ERCC6L2 (Hebo deficiency) |
| *EXTL3* | AR | Immunoskeletal dysplasia with neurodevelopmental abnormalities (EXTL3 deficiency) |
| *FAT4* | AR | Hennekam-lymphangiectasialymphedema syndrome |
| *FCHO1* | AR | FCHO1 deficiency |
| *FOXN1* | AR/AD | Winged helix nude FOXN1 deficiency/FOXN1 haploinsufficiency |
| *GATA2* | AD | GATA2 deficiency |
| *GFI1* | AD | GFI1 deficiency (SCN2) |
| *GINS1* | AR | GINS1 deficiency |
| *HELLS* | AR | Immunodeficiency with centromeric instability and facial anomalies (ICF type 4) |
| *IKBKB* | AD GOF | EDA-ID due to IKBKB GOF mutation |
| *IL2RA* | AR | CD25 deficiency |
| *IL2RG* | XL | γc deficiency (common gamma chain SCID, CD132 deficiency) |
| *IL7R* | AR | IL7Rα deficiency |
| *ITK* | AR | ITK deficiency |
| *ITPKB* | AR | ITPKB deficiency |
| *JAK3* | AR | JAK3 deficiency |
| *LAT* | AR | LAT deficiency |
| *LCK* | AR | LCK deficiency |
| *LCP2* | AR | SLP76 deficiency |
| *LIG1* | AR | Ligase I deficiency |
| *LIG4* | AR | DNA ligase IV deficiency |
| *LRBA* | AR | LPS Responsive Beige-Like Anchor Protein |
| *MAGT1* | XL | X-linked magnesium EBV and neoplasia (XMEN) |
| *MALT1* | AR | MALT1 deficiency |
| *MAN2B2* | AR | MAN2B2 deficiency |
| *MAP3K14* | AR | NIK deficiency |
| *MCM10* | AR | MCM10 deficiency |
| *MSN* | XL | Moesin deficiency |
| *MTHFD1* | AR | Methylene-tetrahydrofolate dehydrogenase 1 (MTHFD1) deficiency |
| *MYSM1* | AR | MYSM1 deficiency |
| *NBS1* | AR | Nijmegen breakage syndrome |
| *NCKAP1L* | AR | NCKAP1L deficiency |
| *NHEJ1* | AR | Cernunnos/XLF deficiency |
| *NSMCE3* | AR | NSMCE3 deficiency |
| *PAX1* | AR | PAX1 deficiency |
| *PGM3* | AR | PGM3 deficiency |
| *PIK3CD* | AR | p110δ deficiency |
| *PNP* | AR | Purine nucleoside phosphorylase deficiency |
| *POLD1* | AR | Polymerase δ deficiency |
| *POLD2* | AR | Polymerase δ deficiency |
| *POLE2* | AR | POLE2 (Polymerase ε subunit 2) deficiency |
| *PRKDC* | AR | DNA PKcs deficiency |
| *PTPRC* | AR | CD45 deficiency |
| *RAC2* | AD GOF | Activated RAC2 defect |
| *RAG1* | AR | RAG deficiency |
| *RAG2* | AR | RAG deficiency |
| *RASGRP1* | AR | RASGRP1 deficiency |
| *RFX5* | AR | MHC class II deficiency group C |
| *RFXANK* | AR | MHC class II deficiency group B |
| *RFXAP* | AR | MHC class II deficiency group D |
| *RHOH* | AR | RHOH deficiency |
| *RIPK1* | AR | RIPK1 |
| *RMRP* | AR | Cartilage hair hypoplasia |
| *SASH3* | XL | SASH3 deficiency |
| *SEMA3E* | AD | CHARGE syndrome |
| *SLC46A1* | AR | SLC46A1/PCFT deficiency causing hereditary folate malabsorption |
| *SMARCAL1* | AR | Schimke Immuno-osseous dysplasia |
| *SOCS1* | AD | SOCS1 haploinsufficiency |
| *STAT1* | AD LOF | STAT1 deficiency |
| *STAT3* | AD GOF | STAT3 GOF mutation |
| *STAT5B* | AR | STAT5b deficiency |
| *STK4* | AR | STK4 deficiency |
| *TAP1* | AR | MHC class I deficiency |
| *TAP2* | AR | MHC class I deficiency |
| *TAPBP* | AR | MHC class I deficiency |
| *TBX1* | AD | TBX1 deficiency |
| *TPP2* | AR | Tripeptidyl-Peptidase II Deficiency |
| *TTC7A* | AR | Immunodeficiency with multiple intestinal atresias |
| *WAS* | XL | Wiskott-Aldrich syndrome (LOF) |
| *WIPF1* | AR | WIP deficiency |
| *ZAP70* | AR | ZAP-70 deficiency (ZAP70 LOF)/ZAP-70 combined hypomorphic and activating mutations |
| *ZBTB24* | AR | Immunodeficiency with centromeric instability and facial anomalies (ICF type 2) |

AD, autosomal dominant; AR, autosomal recessive; GOF, gain of function; LOF, loss of function; MOI, mode of inheritance; XL, X-linked.
